# 401. Risk of Post-COVID Conditions Correlates with IL-6 Plasma Levels and Is Reduced by Early Antibody Therapy

**DOI:** 10.1093/ofid/ofad500.471

**Published:** 2023-11-27

**Authors:** Yuriko Fukuta, Kelly Gebo, Sonya L Heath, Xianming Zhu, Sheriza Baksh, Alison G Abraham, Feben Habtehyimer, David Shade, Jessica E Ruff, Malathi Ram, Oliver Laeyendecker, Reinaldo Fernandez, Eshan U Patel, Owen R Baker, Shmuel Shoham, Edward R Cachay, Judith S Currier, Jonathan Gerber, Barry Meisenberg, Laura Hammitt, Donald Forthal, Moises A Huaman, Adam C Levine, Giselle Mosnaim, Bela Patel, James Paxton, Jay S Raval, Catherine Sutcliffe, Shweta Anjan, Thomas Gniadek, Seble Kassaye, Janis E Blair, Karen Lane, Nichol McBee, Amy Gawad, Piyali Das, Sabra L Klein, Andrew Pekosz, Evan Bloch, Daniel Hanley, Arturo Casadevall, Aaron Tobian, David Sullivan

**Affiliations:** Baylor College of Medicine, Houston, Texas; Johns Hopkins, Baltimore, MD; University of Alabama @ Birmingham, Birmingham, Alabama; Johns Hopkins University, Baltimore, Maryland; Johns Hopkins University, Baltimore, Maryland; University of Colorado, Aurora, Colorado; Johns Hopkins University, Baltimore, Maryland; Johns Hopkins University, Baltimore, Maryland; Johns Hopkins University, Baltimore, Maryland; Johns Hopkins University, Baltimore, Maryland; Johns Hopkins University, Baltimore, Maryland; Johns Hopkins School of Medicine, Baltimore, Maryland; Johns Hopkins Bloomberg School of Public Health, Baltimore, Maryland; The Johns Hopkins University School of Medicine, Baltimore, Maryland; Johns Hopkins University School of Medicine, Baltimore, Maryland; University of California, San Diego, San Diego, CA; David Geffen School of Medicine at University of California, Los Angeles, California; University of Massachusetts, Worchester, Massachusetts; Johns Hopkins University, Baltimore, Maryland; Johns Hopkins School of Public Health, Baltimore, Maryland; University of California, Irvine, Irvine, California; Division of Infectious Diseases, University of Cincinnati, Cincinnati, USA, Cincinnati, OH; Brown University, Providence, Rhode Island; Northshore University Health System, Evanston, Illinois; University of Texas Health Science Center, Houston, Texas; Wayne State University School of Medicine, Detroit, Michigan; University of New Mexico, Albuquerque, New Mexico; Johns Hopkins, Baltimore, MD; University of Miami Miller School of Medicine and Miami Transplant Institute, Jackson Health System, Miami, Florida; Northshore University Health System, Evanston, Illinois; Georgetown University Medical Center, Washington, DC, Washington, DC; Mayo Clinic Hospital, Phoenix, Arizona; Johns Hopkins University, Baltimore, Maryland; Johns Hopkins University, Baltimore, Maryland; Johns Hopkins University, Baltimore, Maryland; Johns Hopkins University, Baltimore, Maryland; Johns Hopkins Bloomberg School of Public Health, Baltimore, Maryland; Johns Hopkins University, Baltimore, Maryland; Johns Hopkins University, Baltimore, Maryland; Johns Hopkins University, Baltimore, Maryland; Johns Hopkins University, Baltimore, Maryland; Johns Hopkins University, Baltimore, Maryland; Johns Hopkins University, Baltimore, Maryland

## Abstract

**Background:**

Post-COVID conditions (PCC) are common, and risk factors include older age and female sex. While high interleukin (IL)-6 and C-reactive protein are associated with more severe disease, it is unclear whether other cytokines are associated with PCC. This study was performed to identify factors associated with PCC development.

**Methods:**

The Convalescent Plasma to Limit SARS-CoV-2 Associated Complications (CSSC-004) trial was a double-blind, multi-center, randomized, controlled trial comparing the use of COVID-19 convalescent plasma (CCP) to control plasma for the prevention of hospitalization among COVID-19 outpatients. Among 882 individuals participating in the trial with available biospecimens and symptom data, the association between early COVID-19 treatment, cytokine levels and PCC was evaluated. Cytokine and chemokine levels were assessed at baseline, day 14 and day 90 using a multiplexed sandwich immuosassay. Presence of any PCC symptom was assessed at day 90. Associations between COVID-19 treatment, cytokine levels and PCC were examined using multivariate logistic regression models.

**Results:**

Baseline characteristics were similar by trial treatment group (Figure 1). At day 90, 292 (33.1%) participants had PCC. The most common symptoms were fatigue (14.5%), anosmia (14.5%), and ageusia (10.0%). Levels of most cytokines decreased over time (Figure 2). Six pro-inflammatory cytokines especially IL-6 were elevated at baseline among those with PCC (Figure 3). In multivariable analysis, female sex (adjusted odds ratio [AOR]=2.70[1.93-3.81]), age 50 or greater (AOR=1.32[1.17-1.50]), and elevated baseline IL-6 (AOR=1.59[1.02-2.47]) were associated with development of PCC, whereas race, obesity, vaccine status and diabetes were not. Those who received early CCP treatment (<5 days after symptom onset) compared to late CCP treatment had statistically significant lower odds of PCC (AOR=0.60 [0.38-0.95]).

**Figure:**
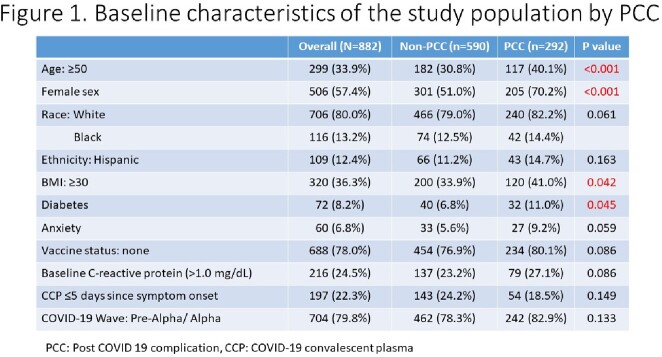

**Figure:**
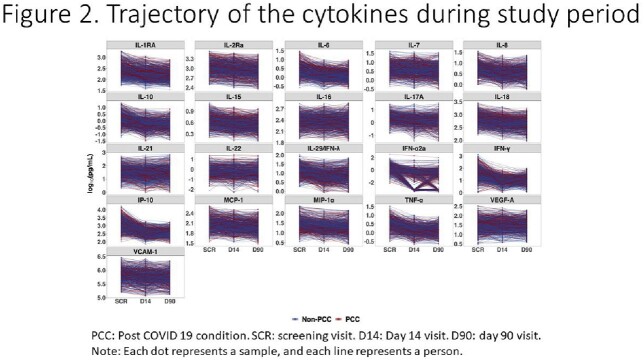

**Figure:**
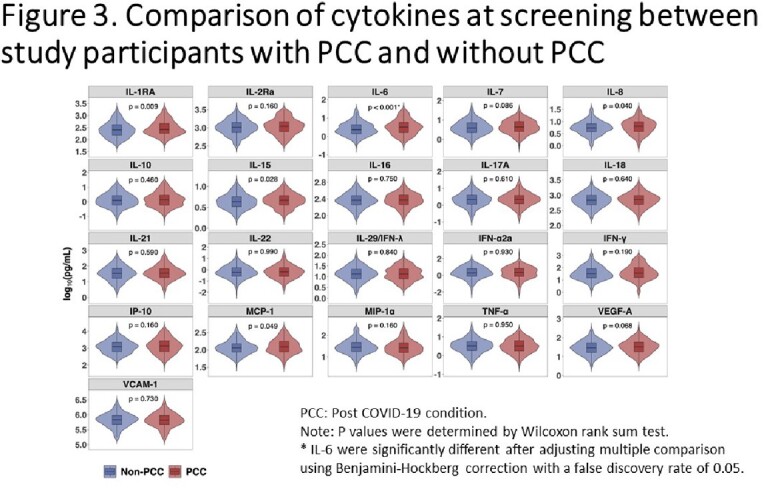

**Conclusion:**

This study showed high prevalence of PCC symptoms at day 90, particularly among those with higher baseline levels of IL-6 or who did not receive early CCP treatment. The potential utility of IL-6 modulation as a therapeutic intervention among individuals as higher risk for PCC should be studied.

**Disclosures:**

**Kelly Gebo, MD, MPH**, Pfizer: Advisor/Consultant|Spark HealthCare: Advisor/Consultant **Sonya L. Heath, MD**, Pfizer: Data Monitoring Committee/DSMB **Shmuel Shoham, MD**, adagio: Advisor/Consultant|Adamis: Advisor/Consultant|ansun: Grant/Research Support|Avir Pharma: Honoraria|cidara: Grant/Research Support|F2G: Grant/Research Support|Immunome: Advisor/Consultant|Karius: Honoraria|Scynexis: Advisor/Consultant|zeteo: Grant/Research Support **Judith S. Currier, M.D., MSc**, Merck and Company: Advisor/Consultant|Merck and Company: Honoraria **Moises A. Huaman, MD, MSc**, AN2 Therapeutics Inc: Grant/Research Support|Gilead Sciences Inc: Grant/Research Support|Insmed Inc: Grant/Research Support **Jay S. Raval, MD**, Sanofi Genzyme: Advisor/Consultant **Arturo Casadevall, MD, PhD**, Ortho Diagnostics: Speakers Bureau|Sabtherapeutics: Advisor/Consultant

